# A novel truncating variant in *PRDM16* causes severe familial cardiomyopathy with variable clinical presentations

**DOI:** 10.1016/j.gendis.2025.101879

**Published:** 2025-10-13

**Authors:** Yuan Che, Lindsey Walker, Lillian Sau, Tingting Tang, Wei Li, Chao Shen, Nick Shillingford, Ramzi Bawab, Jianjun Chen, Miao Sun

**Affiliations:** a Department of Systems Biology and Center for RNA Biology and Therapeutics, Beckman Research Institute of City of Hope, Duarte, CA 91010, USA; b Personalized Care, Department of Pathology and Laboratory Medicine, Children's Hospital Los Angeles, Los Angeles, CA 90027, USA; c Department of Pathology and Laboratory Medicine, Children's Hospital Los Angeles/Keck School of Medicine of USC, Los Angeles, CA 90027, USA; d Division of Genomic Medicine, Department of Pathology and Laboratory Medicine, Children's Hospital Los Angeles/Keck School of Medicine of USC, Los Angeles, CA 90027, USA

Here we report a 10-year-old girl diagnosed with dilated cardiomyopathy, decompensated heart failure, and evidence of global end-organ hypoperfusion in the setting of severely depressed biventricular systolic function. Exome sequencing revealed a novel germline heterozygous frameshift variant resulting from a 2-bp deletion, c.1496_1497del (p.Pro499Leufs∗104) in the PR-domain containing 16 (*PRDM16*) gene, functionally proven to result in PRDM16 deficiency, evidenced by pretermination of PRDM16 protein. Furthermore, the consistent presence of the *PRDM16* variant in affected family members, and its absence in non-affected family members, combined with a strong maternal family history of cardiomyopathy, provides compelling support for the pathogenicity of the *PRDM16* variant. Nevertheless, there is no obvious sex bias in our reported family in terms of *PRDM16*-related cardiomyopathy. We showed that this variant led to impaired mitochondrial function and ATP production. Based upon the collective results of functional studies, clinical features, and family history, this novel heterozygous frameshift germline variant in *PRDM16* was determined to be the cause of the familial cardiomyopathy and heart failure. This novel pathogenic *PRDM16* variant has potential utility in diagnosis and prognosis and underscores the critical and unique role of *PRDM16* in human cardiomyopathy.

PRDM16 is a zinc-finger transcription regulator and has been suspected to be responsible for the cardiomyopathy features in the 1p36 deletion syndrome.^1^ Recent retrospective studies showed that about 29%–34% of patients with a *PRDM16* deletion developed cardiomyopathy, compared with about only 10% of patients without the deletion.^2^

A 10-year-old girl of African American and Mexican ancestry, who was previously healthy with no developmental concerns, was brought to the emergency department due to shortness of breath and persistent cough. Echocardiography in the emergency department revealed a dilated left ventricle (LV) with severely depressed LV systolic function, with an estimated LV ejection fraction 17%–18%. She was subsequently diagnosed with dilated cardiomyopathy, decompensated heart failure, and evidence of global end-organ hypoperfusion in the setting of severely depressed biventricular systolic function. She was admitted for further workup while her heart failure progressively worsened, and she received a heart transplant.

Pathological examination of the explanted heart revealed cardiomegaly (449 g), biventricular mural thickening, diffuse cardiomyocyte hypertrophy in both ventricles, and focal interstitial fibrosis involving the interventricular septum (Fig. 1A). The coronary arteries were patent and within normal limits, with no evidence of inflammation or viral cytopathic changes.

**Figure 1:**
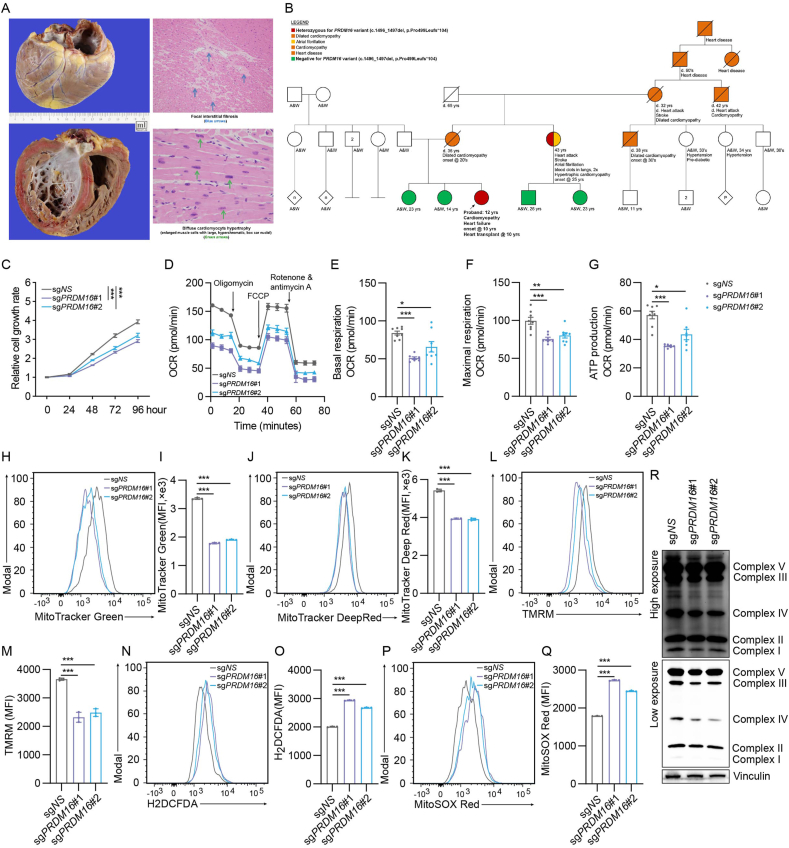
PRDM16 deletion causes cell growth deficiency and mitochondrial dysregulation. **(A)** Pathological examination of the explanted heart of the patient (proband). **(B)** Extended pedigree of the family segregating the heterozygous *PRDM16* frameshift variant (c.1496_1497del, p.Pro499Leufs∗104). **(C)** Cell growth detection after *PRDM16* deletion in 293T cells. **(D**–**G)** Effects of PRDM16 deletion on mitochondrial functions were evaluated by measuring the oxygen consumption rate (OCR) using the Seahorse XFe Extracellular Flux Analyzers. **(H, I)** Mitochondrial mass detection using MitoTracker Green after PRDM16 deletion. **(J, K)** Mitochondrial membrane potential detection by the MitoTracker Deep Red staining. **(L, M)** Mitochondrial membrane potential (ΔΨm) detection by TMRM staining (200 nM) after PRDM16 deletion. **(N**–**Q)** Cellular and mitochondrial ROS level detection by H2DCFDA and MitoSox Red staining (5 μM). **(R)** Detection of mitochondrial respiratory complexes after PRDM16 deletion.

Exome sequencing identified a novel heterozygous frameshift variant, a 2-base-pair (2-bp) deletion (NM_022114.4:c.1496_1497del, p.Pro499Leufs∗104) in the *PRDM16* gene in the patient's peripheral blood (Fig. 1B, red fill; Fig. S1; Table S1). This variant was not detected in her healthy father's blood sample. Her mother is unavailable for genetic testing, as she had passed away from congestive heart failure due to a similar dilated cardiomyopathy phenotype, with symptom onset in her twenties (Fig. 1B; Table S1). An identical heterozygous variant was detected in her maternal aunt, who reported having hypertrophic cardiomyopathy and atrial fibrillation diagnosed at the age of 25 years (Fig. 1B, red and orange fill; Fig. S1; Table S1).

Subsequent genetic counseling revealed an extensive maternal family history with onset of cardiac symptoms in adulthood and subsequent heart failure in multiple generations, including both affected male and female relatives (Fig. 1B, orange fill; Table S1). Further Sanger sequencing analysis completed on available unaffected individuals in the family confirmed the absence of the *PRDM16* frameshift variant (Fig. 1B, green fill). Except for the maternal aunt, all other affected relatives have passed away, and no DNA is available for testing. The consistency with segregation of the variant and the disease in the family suggests this novel *PRDM16* germline variant is likely responsible for the family's extensive history of cardiomyopathy and heart failure.

This 2-bp deletion occurs in exon 9 of the *PRDM16* gene, which is nearly the middle of the whole gene, and is predicted to result in a truncated protein with potential loss of PRDM16 function. Loss-of-function variants in *PRDM16* are highly constrained with pLI = 1 (o/e: 0.21; confidence interval: 0.15–0.3) based on the variants observed in the gnomAD, the largest population allele frequency database (https://gnomad.broadinstitute.org/). To date, this variant has not been linked to any diseases in humans.

A recent study of the patient cohort with PRDM16 loss in the setting of 1p36 deletion syndrome suggests that PRDM16 loss may be associated with sex-dependent cardiomyopathy and cardiac mortality.^2^ In contrast, our patient's family shows no obvious sex predilection, with both male and female members being affected with variable presentations of cardiomyopathy. Moreover, no clear molecular basis was established for this observation in humans. Instead, we hypothesize that loss of PRDM16 in hearts may lead to mitochondrial dysfunction, as suggested in a mouse model.^3^

To evaluate PRDM16's role in cellular growth and mitochondrial function, we first generated *PRDM16*-deletion HEK293T cells using two independent CRISPR–Cas9 sgRNAs targeting the *PRDM16* locus. Western blotting confirmed efficient depletion of PRDM16 protein by both sgRNA sets (Fig. S2A). Compared with the control cells, PRDM16-deficient HEK293T cells displayed a marked reduction in proliferation (Fig. 1C). Given PRDM16's reported involvement in mitochondrial homeostasis,^3^ we hypothesized that this growth defect might arise from mitochondrial dysfunction. We therefore performed Seahorse extracellular flux analysis to measure oxygen consumption rate. As expected, PRDM16 depletion led to a significant decrease in basal respiration, maximal respiration, and ATP-linked respiration (Fig. 1D–G), indicating global impairment of oxidative phosphorylation (OXPHOS) in *PRDM16*-deletion cells. Mitochondrial OXPHOS dysregulation could potentially influence the normal function or biogenesis of mitochondria.^4^ Indeed, *PRDM16* deletion resulted in a significant down-regulation of mitochondrial mass (Fig. 1H and I), significantly decreased the mitochondrial membrane potential (Fig. 1J–M), and significantly increased the mitochondrial and cellular ROS production (Fig. 1N–Q). Mitochondrial OXPHOS is driven by the electron transport chain complexes (ETC).^5^ Our immunoblot analysis showed a marked down-regulation of Complex III and Complex IV of the ETC complexes (Fig. 1R), consistent with findings in Prdm16-deficient murine cardiomyocytes.^3^

To distinguish the functions of full-length versus truncated PRDM16 protein, we cloned and overexpressed the wild-type PRDM16 (WT) and the mutant containing the 2-bp deletion (MUT) in HEK293T cells. The mutant appeared at a lower molecular weight due to premature termination (Fig. S2B). Overexpression of the WT, but not MUT, significantly enhanced cell growth compared with the control (Fig. S2C). Seahorse analysis similarly showed that only the WT overexpression led to increased oxygen consumption rate (Fig. S2D–S2H). Moreover, overexpression of the WT, but not MUT, resulted in significantly increased mitochondrial mass, membrane potential, and lower ROS levels compared with the control (Fig. S2I–S2P). Together, these findings indicate that full-length PRDM16 is essential for maintaining mitochondrial function and supporting cell proliferation, whereas the mutant loses these functions.

In summary, our exome findings of the heterozygous loss-of-function *PRDM16* variant (p.Pro499Leufs∗104) combined with the inheritance, extensive family history of cardiac failure, and functional defects in mitochondria after *PRDM16* deletion, provide evidence that PRDM16 loss of function causes cardiomyopathy and heart failure with variable presentations and ages of onset. Because our assays used HEK293T instead of cardiac models, additional studies in cardiac-relevant systems are warranted to further clarify the variant's biological impact. Elucidation of pathophysiological pathways involving PRDM16 in human heart development and maintenance of normal function is essential for developing drugs targeting *PRDM16*-related cardiomyopathy. The patient is 12 years old now and has been stable after undergoing the heart transplant. She was followed up by the cardiac specialists, geneticists, and other important care management teams.

## CRediT authorship contribution statement

**Yuan Che:** Writing – review & editing, Writing – original draft, Investigation. **Lindsey Walker:** Writing – review & editing, Investigation. **Lillian Sau:** Investigation. **Tingting Tang:** Investigation. **Wei Li:** Investigation. **Chao Shen:** Investigation. **Nick Shillingford:** Investigation. **Ramzi Bawab:** Investigation. **Jianjun Chen:** Writing – review & editing, Writing – original draft, Supervision, Conceptualization. **Miao Sun:** Writing – review & editing, Writing – original draft, Validation, Supervision, Project administration, Investigation, Formal analysis, Data curation, Conceptualization.

## Ethics declaration

This case report is based on a review of clinical records and does not constitute human subjects research requiring formal Institutional Review Board (IRB) approval at Children's Hospital Los Angeles and Keck School of Medicine of USC. Written informed consent for publication was obtained from the patients (or legally authorized representative) for the use of their anonymized medical information and any accompanying images in this report, in accordance with the Guide for Authors for *Genes & Diseases*. Every effort has been made to protect the patient's privacy and ensure anonymity, in accordance with applicable guidelines and best practices regarding patient confidentiality. The patient was informed that, while reasonable efforts were made to anonymize the case report, complete anonymity cannot be guaranteed. A copy of the signed consent form is securely archived and can be made available to the journal or ethics committee upon request.

## Conflict of interests

J.C. is one of Associate Editors for *Genes & Diseases* and was not involved in the editorial review or the decision to publish this article. The authors declare no conflict of interests.
