# Supplementary material for: A novel truncating variant in PRDM16 causes severe familial cardiomyopathy with variable clinical presentations
Source: Genes Dis. 2025 Oct 13;13(3):101879. doi: 10.1016/j.gendis.2025.101879 (PMC12830190; doi:10.1016/j.gendis.2025.101879)
Supplement: Multimedia component 1 [file mmc1.docx]

**Supplementary Data**

**Supplemental Tables**

**Table S1.** Clinical information related to cardiac conditions for the affected family members (See family pedigree Fig. 1B)

| **Family member** | **Clinical information related to cardiac conditions** |
| --- | --- |
| Proband | Onset at 10 years old, dilated cardiomyopathy (LV dilation with severely depressed LV systolic function with estimated LV ejection fraction 17-18%, heart failure, and received a heart transplant. A germline heterozygous frameshift variant (c.1496_1497del, p.Pro499Leufs*104) in the *PRDM16* gene was identified by exome sequencing and confirmed by Sanger sequencing. She is currently 12 years old and is doing well. She is stable from a cardiac standpoint. |
| Mother | Onset in her 20’s, dilated cardiomyopathy and congestive heart failure. She had a pacemaker. She was listed for a heart transplant but did not receive it. She passed away due to cardiogenic shock due to cardiomyopathy at 36 years old. |
| Maternal aunt | Onset at 25 years old, hypertrophic cardiomyopathy and atrial fibrillation s/p cardiac ablation, history of heart attack, blood clots in her lungs 2x, and stroke. She is currently 45 years old. She had discussed with her cardiologist about a pacemaker or ICD placement previously, but no recent update on her treatment since the diagnosis of *PRDM16* mutation (A germline heterozygous frameshift variant, c.1496_1497del, p.Pro499Leufs*104, in the *PRDM16* gene was identified by exome sequencing and confirmed by Sanger sequencing). |
| Maternal uncle | Onset in his late 20’s early 30’s, dilated cardiomyopathy, died from stroke, pneumonia, heart failure, and cardiogenic shock at 38 years old. |
| Maternal grandmother | Dilated cardiomyopathy, cardiomegaly, stroke, and died from a heart attack at 32 years old. |
| Maternal great-uncle | Cardiomyopathy, died from a heart attack at 42 years old. |
| Maternal great-grandfather | Heart disease, died in his late 60’s. |
| Maternal great-aunt | Died from heart disease (No detailed information available) |
| Maternal great-great grandfather | Died from heart disease (No detailed information available) |

**Supplemental Figures**

**Figure S1.** Sanger-sequencing confirmation of the exome finding of the mutation in the *PRDM16* gene in our patient (proband) and her maternal aunt. Top panel shows the sequences of *PRDM16* exon 9 at the mutation site from the forward strand in the normal control 1, normal control 2, maternal aunt, healthy father and proband; the bottom panel shows the reverse sequences correspondingly. Reference sequence is NM_022114.4.


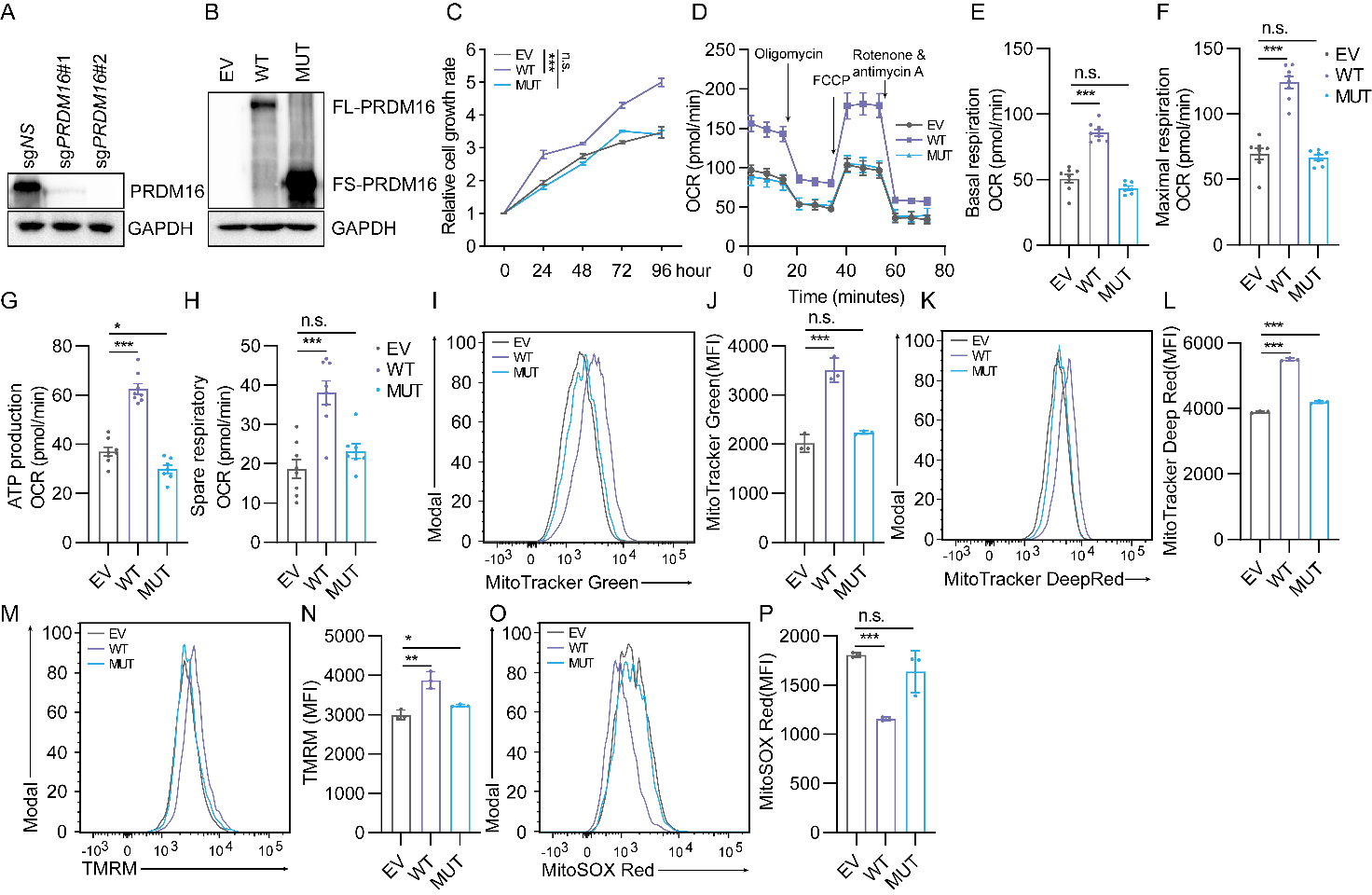


**Figure S2.** Mitochondrial function detection in wild type or mutant PRDM16 overexpressed cells. (A) The knockout efficiency of PRDM16 sgRNAs in HEK293T cells. (B) The overexpression efficiency validation of wild type (full-length, FL) and mutant (MUT, frameshift (FS)) PRDM16 protein in HEK293T cells. (C) Cell growth was monitored in WT and MUT PRDM16 overexpressed cells compared to the control group (EV). (D-H) Effects of WT and MUT PRDM16 overexpression on mitochondrial functions were evaluated by measuring the oxygen consumption rate (OCR) using the Seahorse XFe Extracellular Flux Analyzers. (I, J) Mitochondrial mass detection using MitoTracker Green after PRDM16 overexpression. (K, L) Mitochondrial membrane potential detection by the MitoTracker Deep Red staining. (M, N) Mitochondrial membrane potential (ΔΨm) by TMRM staining (200 nM) after PRDM16 overexpression. (O, P) Mitochondrial ROS level detected by MitoSox Red staining (5 μM).

**Supplemental Material and Methods**

*Cell lines*

Human parental HEK293T cells (obtained from America Type Culture Collection, ATCC) were cultured in Dulbecco’s modified Eagle’s medium, supplemented with 10% fetal bovine serum (FBS), 1% HEPES and 1% penicillin-streptomycin. To get the 293T-Cas9 single clones, a previously established protocol was followed^1^. In detail, parental HEK293T cells were transduced with CRISPR-Cas9 lentivirus followed by the selection step using 10 μg/ml blasticidin treatment. Single cell suspension was seeded into 96 well plates after selection to pick up the single clones. The editing efficiency was confirmed by the CRISPR-Cas9 protein expression.

*Exome sequencing and analysis*

Genomic DNA was extracted from the peripheral blood of the patient (proband), father, and maternal aunt using a commercial kit - Promega Maxwell RSC DNA Extraction Kit (Madison, WI, USA). The Exome Sequencing library was generated using the Agilent SureSelect Human All Exon V6 plus a custom mitochondrial genome capture kit. Captured DNA fragments were then sequenced using the Illumina Nextseq 500 or HiSeq 4000 sequencing system, with 2x100 base-pair (bp) paired-end reads. Single nucleotide variants, and small insertions and deletions (<10 bp) were detected by mapping and comparing the DNA sequences with the human reference genome (GRCh38). To identify the potential disease-causing variants, a primary gene list was generated based on the phenotype-related keywords provided by the physician for prioritization of variant analysis and interpretation. The rare nuclear DNA variants (minor allele frequency <1%) within protein-coding regions and splice-site junctions (5 bp into introns) and any rare mitochondrial DNA variants with <0.5% MitoMap GB frequency were further annotated and analyzed using a commercial tool Agilent Alissa Interpret 5.4 (Santa Clara, CA, USA). Sequence variant classification and interpretation is based on ACMG/AMP standards and guidelines^2^. Variant confirmation by Sanger sequencing was performed for all insertions and deletions as well as substitutions that did not meet the laboratory’s coverage and quality score thresholds (Score < 500 and read depth < 10).

*PRDM16 plasmids*

The wild type PRDM16 was ordered from Addgene (#141659, Addgene). 1496_1497del mutation was generated by site directed mutagenesis of wild type human PRDM16 vector using In-Fusion Snap Assembly cloning kits (638944, Takara). The sgRNAs against PRDM16 was designed using CRISPick (https://portals.broadinstitute.org/gppx/crispick/public) and synthesized by Integrated DNA Technologies (IDT). The sgRNAs were then cloned into lentiGuide-puro plasmid using T4 DNA Ligase (New England Biolabs, M0202T). Correct insertion and sequence fidelity were confirmed by Sanger sequencing. After confirmation, the plasmids were extracted using the Hi-Speed Mini Plasmid Kit (IBI Scientific IB47102).

*Cell proliferation assays*

The cell proliferation assay was assessed by MTT following the manufacturer’s protocols. In detail, PRDM16 wild type or mutant/knockout cells were seeded into a 96-well plate in at least triplicates at the density of 2000 cells/well at day 0. MTT Dye solution was added (10 ul/well) at indicated time points and incubated at 37 ℃ for 3-4 hours before adding the stop solution to end the reaction. The OD570nm was recorded by BioTek microplate reader and all the results were normalized to day 0.

*Lentivirus transduction*

Lentivirus transduction to induce the PRDM16 overexpression or knockout in HEK293T or 239T-Cas9 cells were performed following the laboratory owned protocols^1^. In detail, 5 mg pMD2.G plasmid (Addgene), 5 mg psPAX2 plasmid (Addgene), and 5 mg construct for overexpression or knockout of PRDM16 were co-transfected into HEK293T cells in a 100 mm cell culture dish with Effectene Transfection Reagent (301427, QIAGEN). The virus particles were harvested at 48 and 72 hours after transfection and concentrated with PEG-it virus precipitation solution (LV810A-1, SBI). For transduction, the concentrated virus was directly added into cells in the presence of 8 ug/ml polybrene (H9268, Sigma-Aldrich) and incubated with cells overnight followed by culture medium change. Positively transduced cells were selected with 1 ug/ml puromycin (P8833, Sigma-Aldrich) and expanded for downstream experiments.

*Seahorse analysis*

Cellular oxygen consumption rates (OCR) of PRDM16 overexpressed or knockout 293T cells were detected by Seahorse XFe-96 analyzer (Agilent) following the manufacturer’s instructions. Briefly, a utility plate containing calibrant solution (200 uL per well) was prepared one day in advance and placed in a CO_2_-free incubator at 37 degrees overnight. Cells were seeded at a density of 1×10^5^ per well with 180 ul Seahorse XF DMEM medium in a 96-well Seahorse cell culture plate and placed in a CO_2_-free incubator to equilibrate for at least 30 min. An injector plate containing compounds was prepared (For OCR analysis: oligomycin, 1 mM final concentration; FCCP, 1 mM final concentration; rotenone and antimycin, 0.5 mM final concentration) and run utility plate and injector plate for calibration. After that, the utility plate was replaced with the cell culture plate, the basal respiration rate and the ATP production during this procedure was directly exported from the raw data using Wave software (Agilent).

*Mitochondrial ROS detection*

1×10^5^ HEK293T PRDM16-overexpressed or knockout cells were resuspended with 100 ul culture medium and stained with 5 uM MitoSOX Red at 37 degrees for 30 min. Wash the cells with 0.5 mL PBS for three times, then DAPI staining was added before flow cytometry analysis using BD Fortessa X20. Data were analyzed using FlowJo software, and mean fluorescence intensity was quantified indicating of mitochondrial ROS production.

*Mitochondrial membrane potential detection*

1×10^5^ HEK293T PRDM16-overexpressed or knockout cells were resuspended with 100 ul culture medium and stained with 200 nM TMRM or Mitotracker DeepRed at 37 degrees for 30 min. Wash the cells with 0.5 mL PBS for three times, then DAPI staining was added before flow cytometry analysis using BD Fortessa X20. Data were analyzed using FlowJo software, and mean fluorescence intensity was quantified indicating of mitochondrial membrane potential.

*Mitochondrial mass detection*

1×10^5^ HEK293T PRDM16-overexpressed or knockout cells were resuspended with 100 ul culture medium and stained with 100 nM MitoTracker Green at 37 degrees for 30 min. Wash the cells with 0.5 mL PBS for three times, then DAPI staining was added before flow cytometry analysis using BD Fortessa X20. Data were analyzed using FlowJo software, and mean fluorescence intensity was quantified indicating of mitochondrial mass.

*Western blotting*

Cells were washed and collected with ice-cold PBS and lysed using RIPA buffer for 30 minutes on ice (Pierce, Rockford, IL) containing 5mM EDTA, PMSF, cocktail proteinase inhibitors, and phosphatase inhibitor cocktail. Cell lysis was then centrifuged at 12000 x g for 10 min and the supernatants were added loading buffer and heated at 95 degree for 10 minutes. Antibodies used for Western blot were as follows: PRDM16 (720206, Thermo), OXPHOS Human WB Antibody Cocktail (ab110411, abcam), Vinculin (sc-25336, Santa Cruz Biotechnology), GAPDH (10494-1-AP, Proteintech). Vinculin and GAPDH were used as a loading control.

*Ethic approval*

This case report is based on a review of clinical records and does not constitute human subjects research requiring formal Institutional Review Board (IRB) approval at Children's Hospital Los Angeles and Keck School of Medicine of USC. Written informed consent for publication was obtained from the patients (or legally authorized representative) for the use of their anonymized medical information and any accompanying images in this report, in accordance with the Guide for Authors for *Genes & Diseases*. Every effort has been made to protect the patient's privacy and ensure anonymity, in accordance with applicable guidelines and best practices regarding patient confidentiality. The patient was informed that, while reasonable efforts were made to anonymize the case report, complete anonymity cannot be guaranteed. A copy of the signed consent form is securely archived and can be made available to the journal or ethics committee upon request.

**References**

1. Shen C, Wang K, Li W, et al. A homozygous nonsense mutation in *DNAJC30* causes Leber's hereditary optic neuropathy with Leigh-like phenotypes. *Genes Dis.* 2023;10(4):1165-1168.

2. Richards S, Aziz N, Bale S, et al. Standards and guidelines for the interpretation of sequence variants: a joint consensus recommendation of the American College of Medical Genetics and Genomics and the Association for Molecular Pathology. *Genet Med.* 2015;17(5):405-424.
